# Genetic Dissection of Sorghum Dwarfism Through Systematic Screening of *Dw1*–*Dw3* Alleles in Chinese Germplasm

**DOI:** 10.3390/plants14111703

**Published:** 2025-06-03

**Authors:** Ping Wang, Bingbing Liang, Zhengjun Li, Le Chen, Kejie Liu, Lijuan Wang, Lixia Zhang, Xiaochun Lu

**Affiliations:** 1 Institute of Plant Protection, Liaoning Academy of Agricultural Sciences, Shenyang 110161, China; pingw-556@163.com (P.W.);; 2 Institute of Sorghum, Liaoning Academy of Agricultural Sciences, Shenyang 110161, China

**Keywords:** *Sorghum bicolor*, dwarfing genes, plant height regulation, Chinese landraces, genetic analysis

## Abstract

Sorghum dwarfing genes (*Dw1*, *Dw2*, *Dw3*) are crucial determinants of plant architecture and yield potential; however, their genetic characteristics and distribution patterns in Chinese sorghum landraces remain poorly understood. This study systematically analyzed their allelic distribution across 241 Chinese landrace accessions. Through rigorous PCR-based genotyping and detailed phenotypic characterization, we identified that only approximately 7% of the surveyed landraces carried natural dwarfing alleles, with mutations in the *dw3* locus being the most frequently observed. Plant height statistics and genotyping of F2 plants, whose parents were 8R252 (tall accession) and 8R387 (dwarf accession), demonstrated that *dw3* exerted the most pronounced effect on plant height reduction. Importantly, we discovered significant epistatic effects in double-recessive combinations, with the *dw1dw3* genotype showing particularly strong height reduction. These findings substantially advance our understanding of the genetic architecture underlying sorghum plant height variation and provide a robust scientific foundation for molecular breeding strategies aimed at optimizing lodging resistance and mechanical harvestability in sorghum improvement programs.

## 1. Introduction

Biomass represents a versatile renewable energy source for biofuel production, with feedstock options spanning conventional crops, dedicated energy crops, aquatic biomass, and forestry residues [1,2]. Among these, sorghum (*Sorghum bicolor* (L.) Moench) holds particular importance as a dual-purpose crop. While serving as a dietary staple for approximately 500 million people globally, it has recently gained recognition as a promising bioenergy feedstock [3]. For farmers in semiarid and arid regions, sorghum is an important crop species. Climate change, which leads to rising temperatures and reduced precipitation, is making some areas less suitable for maize and rice production. Consequently, the importance of drought-tolerant crops like sorghum is likely to grow [4].

The breeding of sorghum with dwarfing traits holds great importance. Four major dwarfing genes, namely *Dw1*–*Dw4*, have been identified. These genes control the height of sorghum by modifying the internode length, as discovered by Karper et al. back in 1947 [5]. In 2016, the gene corresponding to *Dw1* was map-based cloned using an F2 population and Heterogeneous Inbred Families (HIFs) derived from Hegari and 80M [6,7]. The *Dw2* gene, located on chromosome 6, can significantly reduce internode lengths due to mutations [8]. *Dw3*, situated on chromosome 7, was cloned decades ago and was found to encode a transmembrane protein of the adenosine [9]. Notably, whereas merely one allelic mutation has been documented for both *dw1* and *dw2*, respectively, the *dw3* locus displays remarkable allelic heterogeneity, with seven different mutant alleles [6,7,9,10,11]. Although *Dw4* has yet to be cloned, it is believed to be located on chromosome 6, according to Morris et al., 2012 [12]. In the early days of hybrid utilization in China, sorghum hybrids were often relatively tall, reaching heights of 2.5–3 m, and this led to serious lodging issues; researchers recognized the need for shorter hybrids (around 2 m in height) for efficient production [13].

To date, no standardized protocol exists for the simultaneous detection of the three major dwarfing genes (*Dw1*–*Dw3*) in sorghum, with significant inconsistencies in primer selection among published studies [6,7,9,10,11]. The development of a robust and streamlined genotyping pipeline is therefore essential, especially to meet the demands of large-scale screening in modern breeding applications.

China is the world’s eighth largest producer of sorghum. Sorghum in China has multiple uses, such as being used as food and feed, for biofuels, and in the production of alcoholic beverages. It was introduced through various means, including the spread of Indian cultivars, during the silkworm trade, or directly from the eastern coast of Africa by Chinese seamen returning home [14,15]. Between the 1930s and 1950s, sorghum was cultivated as a traditional cereal on 10 million hectares. However, the production area saw a sharp decline to around 2 million hectares by 1990 which then further dropped to 0.6 million hectares by 2008 [3]. Instead, sorghum has become increasingly popular as animal feed in recent years, and the Chinese government has invested in research on improved varieties for this purpose, as mentioned by Mundia et al. in 2019 [3]. Moreover, sorghum is a major ingredient in the production of liquor in China’s food industry [16]. Almost all of China’s famous distilled liquor brands, like Moutaijiu (Moutai-aroma liquor), Luzhoulaojiao, Wuliangye (strong-aroma liquor), and Fenjiu (light-aroma liquor), use sorghum grain as a key ingredient in the brewing process, as noted by Zhang et al. in 2023 [17]. Given that sorghum has been planted in China for a long time and boasts rich germplasm resources, it would be beneficial to identify new genes by analyzing the genes that affect plant height in common sorghum cultivars in China. Such corresponding research is highly advantageous for sorghum breeding.

This study systematically analyzed their allelic distribution in Chinese sorghum landraces. Through genotyping and phenotyping of 241 landrace accessions, we characterized the distribution patterns and genetic effects of natural dwarfing alleles. These findings provide new evidence elucidating the genetic basis of plant height variation in sorghum and establish a theoretical foundation for molecular breeding approaches targeting improved plant architecture.

## 2. Results

### 2.1. Dwarfing Gene Detection in 241 Chinese Sorghum Landraces

When utilizing dwarf sorghum materials for dwarfing breeding programs, it is essential to first identify which gene(s) contribute to the dwarf phenotype. Based on the available literature and our team’s previous studies, we established an operating procedure for sorghum dwarfing genes detection (the gene detection methods are described in Section 4, and more detailed operating procedures can be found in Supplementary File S1).

**Figure 1:**
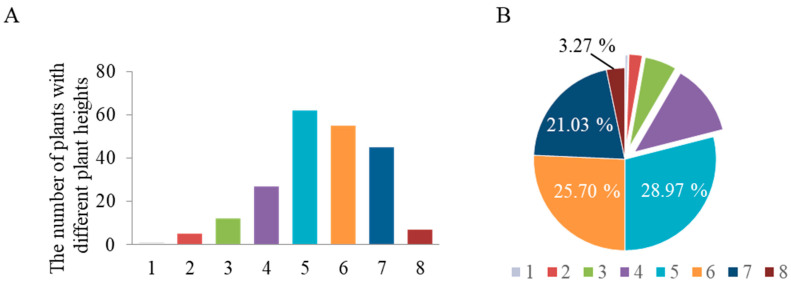
Frequency distribution of plant height in 241 Chinese sorghum landraces. (**A**) shows the statistical distribution of plants with different plant heights. The plants were categorized into eight groups based on height: 1 (0–50 cm), 2 (51–100 cm), 3 (101–150 cm), 4 (151–200 cm), 5 (201–250 cm), 6 (251–300 cm), 7 (301–350 cm), and 8 (351–400 cm). (**B**) displays the percentage distribution of each height group.

China possesses abundant landrace germplasm resources of sorghum for breeders to utilize in targeted breeding programs. However, prior to this study, no systematic investigation had been conducted on the distribution of the three major dwarfing genes (*Dw1*–*Dw3*) in Chinese sorghum landraces. Against this background, our study collected 241 sorghum landraces from across China and cultivated them at the experimental station of the Liaoning Academy of Agricultural Sciences (41°49′18″ N, 123°33′36″ E). At maturity, plant height was measured, and the operating procedure (OP) established by our team was employed to analyze the status of the *Dw1*–*Dw3* genes. The results showed that, as illustrated in Figure 1, the vast majority of sorghum plants exceeded 2.0 m in height, accounting for nearly 80% of the total samples. The broad-sense heritability of sorghum plant height is 93%, indicating that this trait is highly heritable. Genotyping revealed that these materials all carried the genotype *Dw1Dw2Dw3*, indicating a lack of dwarfing genes in traditional Chinese sorghum germplasm.

The genotypes of materials with plant heights below 2.0 m are listed in Table 1. The results demonstrated that, with the exception of 8R617, all accessions carrying at least one of the allelic mutations listed in Table 1 had plant heights below 170.0 cm. This highlights the significant dwarfing effect of the *dw1*–*dw3* genes in Chinese sorghum landraces. Among the 18 accessions carrying at least one of the allelic mutations, 16 involved mutations in the *dw3* gene. Based on plant height observations, all three allelic mutations effectively contributed to reducing sorghum plant height.

### 2.2. Dw3 Plays a Key Role in Sorghum Dwarfing Revealed by 8R252 × 8R387 F2 Population Genotyping

Due to the limited availability of Chinese landrace resources carrying dwarfing genes, as mentioned above, freely utilizing these genetic combinations in breeding programs to apply *Dw1*–*3* genes for height regulation in sorghum is challenging. Given this constraint, it is imperative to elucidate the respective contributions of the *dw1*, *dw2*, and *dw3* genes to sorghum dwarfing, enabling the targeted application of these genes to control plant height within an optimal range.

To address this, we crossed a tall sorghum line (8R252, *Dw1Dw2Dw3*) with a dwarf line from Mexico (8R387, *dw1dw2dw3-ref*) and generated F1 progeny, as presented in Table 2. Self-pollination of the F1 plants yielded 608 F2 individuals. We then genotyped the F2 plants and recorded their plant heights. Subsequent analysis of plant height and genotype data aimed to clarify the individual contributions of *dw1*, *dw2*, and *dw3* to dwarfing.

**Figure 2:**
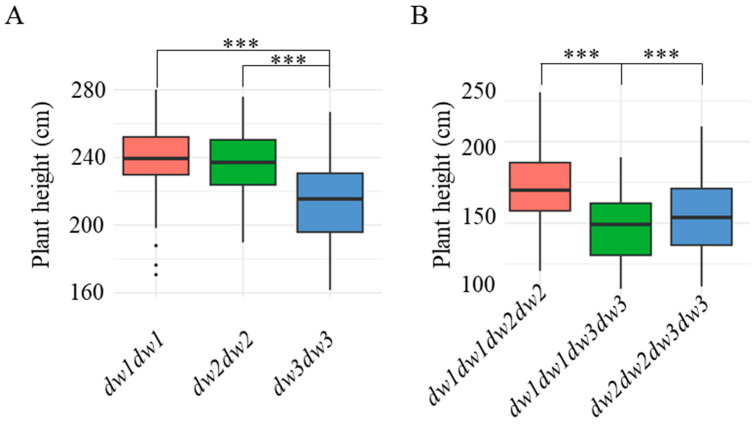
Plant height statistics under single-gene or pairwise-gene homozygous conditions. (**A**) shows the plant height statistics of F2 generation plants when the *dw1*–*3* genes are all single homozygous genes, while (**B**) displays the plant height statistics of F2 generation plants with pairwise recessive homozygous genes. Plant height was investigated (n ≥ 23) in 2022. Asterisks indicate statistically significant differences using Student’s *t*-test (***, *p* < 0.001).

The results revealed that when *dw1*, *dw2*, and *dw3* were homozygous recessive, the average plant heights were 239.5 ± 21.2 cm, 236.5 ± 20.6 cm, and 211.8 ± 24.7 cm, respectively. This indicates that *dw3* exerts a more pronounced effect on height reduction compared to *dw1* and *dw2* when all three genes are homozygous recessive (Figure 2A).

As shown in Table 1, lines 8R337, 8R351, 8R428, and 8R431, which carry two homozygous recessive dwarfing genes, exhibited significantly shorter plant heights. To assess the impact of different recessive allele combinations on dwarfing efficiency, we performed genotype–phenotype association analysis using plant height data from F2 progeny derived from the two parental lines listed in Table 2. The results (Figure 2B) demonstrated that the *dw1dw3* double-recessive combination resulted in the shortest plant height (145.7 ± 20.3 cm), followed by *dw2dw3* (155.2 ± 23.1 cm) and *dw1dw2* (172.6 ± 24.8 cm). This suggests that the *dw1dw3* combination has the most substantial height-reducing effect.

Consistent with this finding, Table 1 shows that lines 8R337, 8R360, and 8R361 (all with the genotype *dw1Dw2dw3-ref*) exhibited plant heights below 1.0 m, whereas 8R351 (*Dw1dw2dw3-ref*) displayed less pronounced dwarfing. These observations further validate our conclusion that the *dw1dw3* combination is particularly effective in height suppression.

## 3. Discussion

Selective breeding for beneficial plant traits is a key strategy for enhancing crop value; it works mainly by optimizing growth and harvest efficiency. In sorghum cultivation, dwarf varieties bring multiple benefits. Their enhanced lodging resistance safeguards the crop against environmental stress, and the reduced plant height simplifies mechanical harvesting operations, as noted by Karper and Quinby in 1947 [5].

Plant height in sorghum is under the control of multiple major dwarfing genes. Quinby and Karper identified four loci (*Dw1*–*Dw4*) that regulate internode length, which directly impacts the plant’s height from the ground to the flag leaf. At each *Dw* locus, the dominant allele promotes internode elongation [6,7,9,10,11]. So far, only three of these four genes (*Dw1*–*3*) have been mapped and cloned, located on chromosomes 9, 6, and 7, respectively [6,7,9,10,11]. The fourth locus, *Dw4*, is known to be unlinked to the others and is hypothesized to be on chromosome 4 or 6, but the associated genes remain uncloned [12,13].

Currently, one allelic mutation has been identified in *dw1* or *dw2*, respectively [6,7], while *dw3* is more extensively studied, with seven allelic mutations reported across multiple studies [9,10,11]. Among the allelic mutations of *dw3*, some involve large insertions, others deletions of 2–82 nucleotides, and some merely single-nucleotide substitutions [9,10,11]. However, the three allelic variants (*dw3*–*sd3*, *dw3*–*sd4*, and *dw3*–*sd5*) were not detected in the Chinese landraces surveyed. This discrepancy may stem from differences in the geographic origins of sorghum germplasm, as these three mutations were originally identified in accessions from the Americas. At present, the identification of allelic mutations relies on PCR amplification followed by Sanger sequencing and comparison with the wild-type sequence. For *dw3* genotyping, different primers have been used in various studies. Our previous research revealed that the primers we designed could amplify products covering all currently known mutation types of *dw3* [10]. Based on these findings, we have summarized and developed a standardized protocol for detecting sorghum dwarfing genes, providing a valuable reference for breeders and facilitating research in this field.

In China, sorghum has been a crucial ingredient in liquor-making since the Yuan Dynasty (1270–1368 CE) [18,19]. It is mainly grown in the southwest, north, and northeast regions of the country, and more than 80% of sorghum grain is used in the liquor industry [18,20]. China’s rich sorghum germplasm resources provide a valuable foundation for new cultivar selection and breeding. Prior to this study, the genetic architecture of plant height in traditional Chinese sorghum landraces remained largely unexplored. Wang et al. (2024) classified the dwarfing genotypes of Chinese male sterile lines and restorer lines, and investigated the effects of genotypic differences on the plant height of hybrid progenies [21]. Our study, together with that of Wang et al. (2024) [21], systematically analyzed the genetic effects and breeding application values of sorghum dwarfing genes *Dw1*–*Dw3*. The two studies complement each other in terms of research objects (local germplasm resources vs. major breeding materials) and research dimensions (distribution of allelic variations vs. genetic effects of hybridization). Both studies jointly confirmed that *Dw3* is a core gene for plant height regulation, and the frequency of dwarfing alleles in Chinese germplasms is significantly low. The two studies, respectively, constructed a theoretical framework for molecular design breeding from the perspectives of germplasm resource innovation (allelic variation mapping) and breeding material selection (genotype–phenotype prediction). The consistent conclusions (dominant effect of *Dw3* and scarcity of dwarfing sources) provide bidirectional evidence for multi-gene pyramiding breeding and promote systematic research on coordinated improvements in plant height and yield.

Notably, the dwarf phenotype, observed in several accessions (including 366, 396, etc.) that maintained wild-type genotypes (*Dw1Dw2Dw3*) at all three major loci, may be explained by the existence of undiscovered minor-effect genes, given that only 3–4 major dwarfing genes have been identified while numerous minor quantitative trait loci affecting plant height remain to be characterized. This phenomenon highlights the genetic complexity underlying sorghum dwarfism and suggests that the current understanding based solely on major genes is incomplete, pointing to the need for more comprehensive genetic studies to uncover additional contributors to plant height variation. The presence of these dwarf accessions with wild-type genotypes at known loci provides valuable materials for future investigations into alternative dwarfing mechanisms and the polygenic regulation of sorghum plant architecture.

Through carefully designed crosses between wild-type (*Dw1Dw2Dw3*) and triple-mutant (*dw1dw2dw3*) lines, coupled with detailed phenotypic analysis of F2 populations, we established clear genotype–phenotype relationships. Our results demonstrate that when *dw3* was completely homozygous recessive, its effect on reducing plant height was the most obvious, while combinations show additive effects on plant height reduction. Among different pairwise combinations, the combination of *dw1dw1dw3dw3* obviously causes the most significant dwarfing of the plants. These findings provide the first quantitative framework for understanding how different allelic combinations influence plant architecture in sorghum.

This research provides significant advancements for sorghum breeding by elucidating the genetic basis of plant height regulation. Through comprehensive genotyping analysis, we have characterized specific allelic effects that facilitate more targeted breeding strategies for height optimization. Notably, the identification of dwarf landraces without known dwarfing gene mutations points to the presence of novel genetic determinants in Chinese sorghum germplasm. These findings not only enhance our fundamental understanding of plant architecture genetics but also offer practical tools for molecular breeding applications. The study bridges the gap between basic genetic research and crop improvement by providing both theoretical insights and actionable breeding knowledge.

## 4. Materials and Methods

### 4.1. The Cultivation of Plant Materials

In 2022, a comprehensive phenotypic evaluation was conducted on 241 Chinese sorghum landraces at the experimental station of Liaoning Academy of Agricultural Sciences (42° N, 123° E). The trial employed a standardized plot design (4 × 2.2 m) with three rows per plot, maintaining 60 cm row spacing and 30 cm intra-row plant spacing. For phenotypic assessment, a minimum of 15 representative plants were sampled from each plot’s central area. Plant height measurements, recorded at maturity from soil surface to apex in autumn 2022, followed an optimized protocol adapted from Zou et al. [22] to ensure data reliability. Subsequently, plant height measurements were replicated in 2023, yielding consistent results with the initial observations.

### 4.2. Detection and Genotyping Methods for dw1–dw3 Genes

#### 4.2.1. Detection of *dw1*

Genomic DNA was isolated from pooled fresh leaf tissues samples (5–6 plants per accession) using the CTAB method. DNA quality control involved dual verification through spectrophotometric analysis (NanoDrop ND-1000, Waltham, MA, USA) and electrophoretic separation on 1.0% agarose gels.

PCR amplification of *Dw1* loci was performed in 25 μL reactions containing the following: 2× EXtaq polymerase (Takara, Otsu, Shiga, Japan), 0.4 μM of each primer, 125 ng template DNA, and nuclease-free water. The thermal cycling conditions were optimized for each target gene.

*Dw1* gene amplification: Initial denaturation at 95 °C for 5 min; 34 cycles of 94 °C for 45 s (denaturation), 56 °C for 45 s (annealing), and 72 °C for 60 s (extension); final extension at 72 °C for 15 min.

Using primers Dw1F and Dw1R (Table 3), a ~427-bp fragment was amplified via PCR [6]. The PCR products were sequenced by Sanger sequencing at Biomarker Technologies Corporation (located in Beijing, China) to obtain the amplified product, and the sequence was aligned with the wild-type *Dw1* gene (Phytozome database ID: Sobic.009G229800; https://phytozome-next.jgi.doe.gov/ (accessed on 1 October 2022)). A thymine (T) at position 1350 of the *Dw1* sequence indicates the *dw1* genotype, whereas other nucleotides correspond to the wild-type *Dw1* [6,7].

#### 4.2.2. Detection of *dw2*

The DNA extraction method and PCR reaction system were consistent with Section 4.2.1, while the amplification and alignment of the *dw2* gene were performed according to the following protocol:

*Dw2* gene amplification: Initial denaturation at 95 °C for 5 min; 34 cycles of 94 °C for 45 s, 58 °C for 45 s, and 72 °C for 60 s; final extension at 72 °C for 15 min.

Primers Dw2F and Dw2R (Table 3) were used to amplify a ~997-bp fragment. Sanger sequencing of the product was compared to the wild-type *Dw2* gene (Phytozome ID: Sobic.006G067700; https://phytozome-next.jgi.doe.gov/ (accessed on 1 October 2022)). The deletion of nucleotides GA at positions 549–550 in the *Dw2* sequence confirms the *dw2* genotype; retention of GA indicates the wild-type *Dw2*.

#### 4.2.3. Detection of *dw3*

This was the same as Section 4.2.1 for DNA extraction and PCR setup. *Dw3* gene-specific amplification and comparison methods were as follows:

*Dw3* gene amplification: Initial denaturation at 95 °C for 5 min; 34 cycles of 94 °C for 45 s, 58 °C for 45 s, and 72 °C for 90 s; final extension at 72 °C for 15 min.

Primers Dw3F and Dw3R (Table 3) were used to amplify the target fragment, which was then aligned with the wild-type *Dw3* gene (Phytozome ID: Sobic.007G163800; https://phytozome-next.jgi.doe.gov/ (accessed on 1 October 2022)). Seven mutation types (*dw3*–*ref*, *dw3*–*a*, *dw3*–*b*, *dw3*–*c*, *dw3*–*sd3*, *dw3*–*sd4*, and *dw3*–*sd5*) were identified (Table 1). The *dw3-ref* mutation yields a ~2165-bp PCR product, while the other six mutations produce ~1280-bp fragments. Except for *dw3-ref*, all variants require Sanger sequencing using primer Dw3F to verify specific mutation sites (alignment positions listed in Table 3).

By following this method, the dwarfing genotypes (*dw1*, *dw2*, and/or *dw3*) of sorghum materials can be systematically determined. The mutation sites of the three dwarfing genes in the sequence are listed in Table 3. For a more detailed methodology, please refer to Supplementary File S1.

### 4.3. Methods for Generating the 8R252 × 8R387 F2 Population and Genotyping

#### 4.3.1. Hybridization Method for Parental Lines

The hybridization of parental plants was conducted in the summer and autumn of 2020. At 1/3 flowering stage of the female parent ear, wed cut off the top 1/2 of the ear, then used tweezers to remove all anthers from each floret before immediately bagging for isolation. Pollination should be conducted within 24–48 h after emasculation by gently tapping the male parent ear to collect fresh pollen, which is then sprinkled onto the stigma of the female parent. We re-bagged immediately after pollination and labeled the hybrid cross clearly. The resulting F1 generation seeds were sown the following year (2021) and we allowed the plants to self-pollinate to produce the F_2_ generation.

#### 4.3.2. Phenotyping and Genotyping of F2 Plants

We sowed the F_2_ seeds in the third year (2022). In the same autumn, plant height was statistically analyzed following the method described in Section 4.1, and genotyping was performed according to the protocol in Section 4.2. For example, homozygous genotypes were designated as *Dw1Dw1* or *dw1dw1*, while heterozygous genotypes were marked as *Dw1dw1*.

### 4.4. Data Analysis

To evaluate genotype–phenotype associations, plants sharing identical genotypes were systematically categorized for comparative analysis. Statistically significant differences in plant height among genotypic groups were assessed using Student’s *t*-test (SPSS version 26; IBM Corp., Armonk, NY, USA).

Broad-sense heritability (H2) was calculated using the formula H2 = VG/VP. VG and VP are the genetic variance and phenotypic variance, respectively.

## 5. Conclusions

We systematically analyzed 241 Chinese landraces, revealing that only 7% carried natural dwarfing alleles, predominantly in *dw3*. The genetic analysis demonstrated *dw3*’s superior effect on height reduction, with the *dw1dw3* combination showing the strongest epistatic dwarfing. This work provides breeders with a reliable molecular toolkit while advancing our understanding of sorghum height regulation.

## Figures and Tables

**Table 1:** Accession numbers, genotypes, and plant height statistics of Chinese landraces (<2 m).

Accession Number	Genotype	Plant Height (cm)	Accession Number	Genotype	Plant Height (cm)
8R241	*Dw1Dw2dw3-c*	46.0 ± 3.5	8R360	*dw1Dw2dw3-ref*	79.7 ± 4.9
8R257	*Dw1Dw2Dw3*	141.0 ± 14.7	8R361	*dw1Dw2dw3-ref*	98.0 ± 10.6
8R258	*Dw1Dw2dw3-c*	129.3 ± 6.0	8R366	*Dw1Dw2Dw3*	121.0 ± 24.1
8R270	*Dw1Dw2dw3-ref*	154.3 ± 17.9	8R396	*Dw1Dw2Dw3*	183.7 ± 9.3
8R272	*Dw1Dw2dw3-ref*	150.3 ± 6.0	8R402	*Dw1Dw2dw3-b*	124.7 ± 5.5
8R274	*Dw1Dw2Dw3*	195.3 ± 15.5	8R414	*Dw1Dw2Dw3*	176.0 ± 12.8
8R275	*Dw1Dw2dw3-ref*	132.3 ± 2.5	8R415	*Dw1Dw2Dw3*	150.7 ± 16.3
8R277	*Dw1Dw2Dw3*	156.7 ± 9.8	8R417	*Dw1Dw2dw3-c*	131.0 ± 5.3
8R281	*Dw1Dw2Dw3*	135.0 ± 5.0	8R428	*Dw1Dw2dw3-c*	169.7 ± 13.1
8R288	*Dw1Dw2Dw3*	174.7 ± 12.7	8R431	*Dw1Dw2Dw3*	100.3 ± 10.3
8R299	*dw1Dw2Dw3*	133.7 ± 8.1	8R462	*Dw1Dw2Dw3*	170.0 ± 21.4
8R315	*Dw1dw2Dw3*	160.3 ± 8.4	8R463	*Dw1Dw2Dw3*	170.0 ± 17.4
8R336	*Dw1Dw2dw3-ref*	134.0 ± 7.2	8R617	*Dw1Dw2dw3-ref*	188.7 ± 8.1
8R337	*dw1Dw2dw3-ref*	71.7 ± 4.7	8R633	*Dw1Dw2dw3-ref*	101.0 ± 11.5
8R351	*Dw1dw2dw3-ref*	128.3 ± 2.3	8R634	*Dw1Dw2dw3-ref*	93.7 ± 5.7

**Table 2:** Phenotype analysis for tall/dwarf plant height.

Cultivar Number	Origin	Genotype	Plant Height (cm)
8R252	China	*Dw1Dw2Dw3*	265.0 ± 11.8
8R387	Mexico	*dw1dw2dw3-ref*	66.3 ± 4.2

**Table 3:** Primers and allelic mutation types for detection of *dw1*–*dw3* genes.

Gene ID in the Database	Allelic Mutation Number	Primers Used for Identification (5′–3′)	Location and Pattern of Allelic Mutations	References
Sobic.009G229800	*dw1*	F:TGGCGGTCCAACGTCTAAT R:CCTGAAGTATGGCGTGTCG	T at position 1350	[6,7]
Sobic.006G067700	*dw2*	F:CAGTTCAAATCAACGAGGAG R:TCCGTCGTGAAATGAGAATA	GA deletion at 549–550	[8]
Sobic.007G163800	*dw3*–*ref*	F:CCGTCATCGTCCAGAACTCG R:CTTGAGCAGGTGCGAGTGCGA	882 bp insertion (6204–7085)	[9]
	*dw3*–*a*		A to C substitution at 5406	[10]
	*dw3*–*b*		A to G substitution at 5668	
	*dw3*–*c*		2-bp deletion at 5967–5968	
	*dw3*–*sd3*		82-bp deletion at 5485–5566	[11]
	*dw3*–*sd4*		6-bp repeat at 5820–5825	
	*dw3*–*sd5*		15-bp deletion at 5997–6011	

The database website: https://phytozome-next.jgi.doe.gov/ (accessed on 1 October 2022). The amplified fragments were compared with the sequences in the database, and the mutation positions refer to the “ATG” start of the wild-type sequence. The gene IDs for *Dw1*, *Dw2*, and *Dw3* are Sobic.009G229800, Sobic.006G067700, and Sobic.007G163800, respectively.

## Data Availability

Data are contained within the article and Supplementary Materials.

## Supplementary Materials

The following supporting information can be downloaded at: https://www.mdpi.com/article/10.3390/plants14111703/s1, File S1: Detection Procedures for Sorghum Dwarfing Genes *Dw1*–*Dw3*.
