# Supplementary material for: Genetic Dissection of Sorghum Dwarfism Through Systematic Screening of Dw1–Dw3 Alleles in Chinese Germplasm
Source: Plants (Basel). 2025 Jun 3;14(11):1703. doi: 10.3390/plants14111703 (PMC12158038; doi:10.3390/plants14111703)
Supplement: Supplementary file 1 [file plants-14-01703-s001.zip › plants-3650598-supplementary.pdf]

Detection Procedures for Sorghum Dwarfing Genes *Dw1-Dw3*

Step 1. Extraction of DNA and Configuration of PCR Reaction System

Genomic DNA was isolated from pooled fresh leaf tissues samples (5-6 plants per accession) using the CTAB method. DNA quality control involved dual verification through spectrophotometric analysis (NanoDrop ND-1000, USA) and electrophoretic separation on 1.0% agarose gels.

PCR amplification of *Dw1* loci was performed in 25 μL reactions containing: 2× EXtaq polymerase (Takara, Japan), 0.4 μM of each primer, 125 ng template DNA, and nuclease-free water. The thermal cycling conditions were optimized for each target gene.

Step 2. Detection of whether the sorghum dwarfism is caused by the dw1 gene

Amplify partial fragments of the *dw1* gene using the the PCR reaction system in step1.

Dw1 gene amplification: Initial denaturation at 95°C for 5 min; 34 cycles of 94°C for 45 s (denaturation), 56°C for 45 s (annealing), and 72°C for 60 s (extension); final extension at 72°C for 15 min.

Using primers Dw1F and Dw1R (Table 1), a ~427-bp fragment was amplified via PCR. The PCR products were sequenced by Sanger sequencing on Biomarker Technologies Corporation (located in Beijing, China). of the amplified product was performed, and the sequence was aligned with the wild-type *Dw1* gene (Phytozome database ID: Sobic.009G229800; https://phytozome-next.jgi.doe.gov/(accessed on October 1st, 2022)). A thymine (T) at position 1350 of the *Dw1* sequence indicates the *dw1* genotype, whereas other nucleotides correspond to the wild-type *Dw1*.

Step 3. Detection of whether the sorghum dwarfism is caused by the dw2 gene

After completing Step 2, proceed to detect the dw2 gene. Similarly, conduct PCR reactions using the reaction system described in Step 1.

Dw2 gene amplification: Initial denaturation at 95°C for 5 min; 34 cycles of 94°C for 45 s, 58°C for 45 s, and 72°C for 60 s; final extension at 72°C for 15 min.

Primers Dw2F and Dw2R (Table 1) were used to amplify a ~997-bp fragment. Sanger sequencing of the product was compared to the wild-type Dw2 gene (Phytozome ID: Sobic.006G067700; https://phytozome-next.jgi.doe.gov/(accessed on October 1st, 2022)). The deletion of nucleotides GA at positions 549–550 in the *Dw2* sequence confirms the *dw2* genotype; retention of GA indicates the wild-type *Dw2*.

Step 3. Detection of whether the sorghum dwarfism is caused by the dw3 gene

Dw3 gene amplification: Initial denaturation at 95°C for 5 min; 34 cycles of 94°C for 45 s, 58°C for 45 s, and 72°C for 90 s; final extension at 72°C for 15 min.

Primers Dw3F and Dw3R (Table 1) were used to amplify the target fragment, which was then aligned with the wild-type *Dw3* gene (Phytozome ID: Sobic.007G163800; https://phytozome-next.jgi.doe.gov/(accessed on October 1st, 2022)). Seven mutation types (*dw3-ref, dw3-a, dw3-b, dw3-c, dw3-sd3, dw3-sd4, and dw3-sd5*) were identified (Table

1). The *dw3-ref* mutation yields a ~2165-bp PCR product, while the other six mutations produce ~1280-bp fragments. Except for *dw3-ref*, all variants require sanger sequencing using primer Dw3F to verify specific mutation sites (alignment positions listed in Table 3).

By following this method, the dwarfing genotypes (*dw1*, *dw2*, and/or *dw3*) of sorghum materials can be systematically determined. The mutation sites of the three dwarfing genes in the sequence are listed in Table 3. For more detailed methodology, please refer to Supplementary File 1.

Table S1. Primers and Allelic Mutation Types for Detection of *dw1-dw3* Genes.

Gene ID in the database	Allelic mutation number	Primers used for identification (5'–3')	Location and pattern of allelic mutations	References
Sobic.009G229800	*dw1*	F:TGGCGGTCCAACGTCTAAT<br>R:CCTGAAGTATGGCGTGTCG	T at position 1350	[6,7]
Sobic.006G067700	*dw2*	F:CAGTTCAAATCAACGAGGAG<br>R:TCCGTCGTGAAATGAGAATA	GA deletion at 549-550	[8]
	dw3-ref		882bp insertion (6204-7085)	[9]
	dw3-a		A to C substitution at 5406	
Sobic.007G163800	*dw3-b*	F:CCGTCATCGTCCAGAACTCG	A to G substitution at 5668	[10]
	dw3-c	R:CTTGAGCAGGTGCGAGTGCGA	2-bp deletion at 5967-5968	
	dw3-sd3		82-bp deletion at 5485-5566	
	dw3-sd4		6-bp repeat at 5820–5825	[11]
	dw3-sd5		15-bp deletion at 5997–6011	

The database website: https://phytozome-next.jgi.doe.gov/ (accessed on 1 October 2022). The amplified fragments were compared with the sequences in the database, and the mutation positions refer to the “ATG” start of the wild-type sequence. The gene IDs for *Dw1*, *Dw2*, and *Dw3* are Sobic.009G229800, Sobic.006G067700, and Sobic.007G163800, respectively.

Appendix：

The following are the wild-type gene sequences of *dw1-dw3*, ranging from the start codon to the stop codon, including exons and introns. These sequences are sourced from the website: https://phytozome-next.jgi.doe.gov/ (accessed on 1 October 2022).

***Wild-type sequence of Dw1*：**

ATGTCTTCAGTTGGGAGCAGCCCTGGCACCAGGGCGGCGAATGGTGCCGCTGCCATCAGTGCTG

CCGCGACGGCAGCAGGCTCAGCCGACGCCAGATTCCACTCCCAACTACTACAGCAGGACAGGG

TACATACACTATATACCACTTGTTGTTCCATGGGCGGATGCAATCGGGCTTGGGCTTTAGCTGCAG

CTTTTGCCTTGCATGCTTTAGATATGTGGACGACAGGATCAGTACCGGAATTTTTAGTCGGCCTTG

ACGACCTAGATTGGCAATTTGGGCCAGCCTTTGCTTACTCAGTACGAATTGTACTGCCCTGCAGT

GGTTATGACTTGAAACTGGGCATCTTTACCAGCAATAATTTGGCTTCAGTACTATATATATGCTTGG
TTGCTTCTGTCAGATAATTTATTTAGTTGGATTTTGCTTTTGCTATGTTGTAATGCACACGTTTTGCA
CTCCTTATTTTGTTTTCAGTTCCAGAGGCTTTTGTGATATCATTCCTCCAGAGAATGTATATACTAAT
AAGTTCTTGATTTTACCCCTCGATACACTCCCACCCATTTGATGTTGAAGTAGGGGCAAAAAGCT
TTTACACCTGGGATAATCCTTGCTTTATGATAAAAATCTTTTTCCATGTGAATACTTTTGGATTTTTG
TGAAACACTAACATTTCGTATGTTCTATTGTATGATTCCTTTGATGCTTATTCTTATGCATTTTTTCTT
CCCACAACACAATGATAATAAAAATGTTTTTTTGTATACAAAGTATAGATTACTGCTCCTTTGAAA
TTTCGTTGTCATGTGTTTTCTTTTATTTCTGTGGTGACAGCTAAGTTATTTGCTTGGACTAACACAC
GCTTCTCTACTCTAAACAGCAAAGTAGATGGGCTGGCTGCTTTTCAGGTTTATCATGTTTCGGATC
GCAGAAGGGCGGGAAAAGAATTGTTCCTGCAGCACGTACTTCTGATGGGAATGGATCAAATGCT
CGTGGAAATGGTCAATCTGGTGCCAATTCAAACCAAAATATGCCTATGAATTTATCTCTTCTGGCT
CCACCATCATCACCAGCATCCTTCTCAAACTCTGCCCTTCCTTCGACTGCTCAATCACCGAATTG
CTTTCTGTCTGTATCTGCAAACTCTCCTGGCGGTCCAACGTCTAATATGTTCGCTGTTGGGCCATAT
GCTAATGAACCTCAACTTGTCTCGCCACCTGTCTTCTCAACTTACACAACTGAGCCATCCACAGC
ACCATTGACCCCACCACCTGAACTAGCTCATGCAACAACGCCTTCTTCTCCAGATGTTCCATATG
CTCGATTTCTTTCTTCTTCTATGGATATCTAAACTGCCAGCAAGGAGCATAACATGCCTTTCTTATC
AACAGCCTATTCTGGTGGTTCAGGACTCCAGGCATCCTACCCACTTTACCCTGAAAGCCCTTGTA
GCAGCCTCATCTCACCAGCTTCAGTAACTCCGAGGACTGGTCTATCCTCACCTATACCTGAGCAA
GAGGTTCCTCCTGCCCATTGGAAGACCTCTAGGTCCGCCTGCGACACGCCATACTTCAGGGCTTC
ACCAATTCCTGAGCAAGAGACCACTGCACAATGGAAGACCTCTAGATCTGCTTGTGACACACCG
TATGCCAGGAACTCACCAACCAATATTTTTGGTCTGGATTCATCTACCCCGAGAAACTATATGCTA
GATAGCAATTTCTTTCGGCCAGCGGCATCTGCTCAATTCTACCTGGACCAGGCTCAGCAGACGTT
TCCACATAATGGCGGGAGGGTTAGTGTATCCAGGGAGAAGCAAGATGCCGATGAAATCGAAGCT
TACAGAGCTTCATTTGGGTTTAGTGCTGATGAAATTGTCCAATCTCAATCTTATGTGGGGATACCA
GATGCGGTTGATGAGTCATTCAGTATATCACCATTTGGAAACAATGCTCCTGCTACTGAGATATGC
CCATTTAGTGATCTGCCTAATGAGGTTCAGAAGGTGGATAAATCATGTGCCTACGCTAAAGATGG
CACAAGTCCAAAGAAATCAGCAAACCAACTCTCCATTGATTCTCCAAATAAAGTTCTGCGCTTG
GACGTGTTCAAAGGTAACATTGGATTTTTCTTGGTACTGTGTGAAATAATAATCTGCTGAATTGAA
ATACATATTGTAGGCTGATTTGTACACCCAGATTTTAATAGTAACAAATTTGTTATGGCCCTACTAT
TGTTGTGTTTCTGTTTCCACATGCTTATTGTGTTTCTGTTCTCAAATGGCAGGAACAAAAGGAGGT
CATCAGTCTGAGGACGAGGGTATTGTGAAAGATGGTCATCCTTTCAGAAGGACAACAGATGAAA
TATCTCTAAAACCCATAGAAGTAAGGAAGAAATCTCTTCCTGGCCATTCTTGCTCAGATGCAGAA
ATCGAGTACAGAAGGACAAGGAGTTTGAGGGATGCCAATGGTGTTTTATCGCGGCGGAGTGCAT
TGGCAAGACAATTGCATTAA

Wild-type sequence of Dw2：

ATGCAGCAGCCGTTCTACGGCCGGCCTCCCACCCAGATGAAGGTGCAGCTCGTGGAACAGGAG
GGCTCCAAGAGGATGGGCTCTTCCGGGTGCTCGGAGATCGTCGAACTGGTGGACGAGCCGAAG
GATGCTCGTCCGGGTGGGCTTACCCACCTGAGGGTGAGGGTGAAGCCAGTGGGGCAGGAGCAT
GGGGCGCGGTCGTGCTCGGTGGAGGATGATCTTGACCAGCTCATCAGATCGATCAACGTGCGCA
CGTCGGCGAGGGCTTCTGGGCAGACGAGCACGGATCGGCGGCTTATTGCGCTCGGGAAGTCGCC
GGTGTCGAGCTCGGAGATTGTGGAGTCTGTGAGCCTGAAGCAGGCCCTGAGGAAGATGTGCATC
TCCCAGGCGTCGGAGATGGCAGCCATGAAGAGGCTTTCGAAGCCGACAGGGGTGTCCACCCCT
TCTGATTCTGGGCCAATCAAGAAGCTATATGGCTCTGTTGCAGTTCAAATCAACGAGGAGCAAG

ATGACAAGAGTAAGGCGGGGAAAGTTTCTATGCTGCCTGAGAAATTGGCAGGAAGTTCAGTGG
GTAAGCCAAGTGGAATCGGTAAGGGGCAGAGCAAGAGCTCGGCCAAGAAGAATTTGAGATCA
GCATCTCCTACCACTGGCAAGGTTCATAAGACCAGAATCCAAGATGTGATCAGTAACAAGTCAT
CAGAGGGAGTTGAAGATATTTCTGTAGGAGCAACACTGCCAAAACAGAGGAAGGGAAAATCTG
TGAAAACATCTAGCCCCCGGGCTGTTCCAGTAGGTGGCTCACGGCTGGTGAGGCCAATGTTTCG
GAATAAAACCTCGACGAAAAAGAAGGTCAAACCTGAACCTGCTATAGTGCCCGCATCACACAA
GCATTGTGAGACGAAAGGTCCTAAATCTCACACTAGTAAACAGCAGGAGCCCTTTCAGGATGAA
CCCAGAACCCCAGCACCGACTAACAAGAAGGCTGCTGTCAGCTCCACCACCGTTGATGGAGCT
GATTTTGGCACCAAAGGATGTGTTGGTGTGATCCATGGCTCAAAGGTTGGTGAATTGTCGAGATC
AAAGGAGAAGGGCGAATGCTCCCAAAGCTCTAAGAGTAGCATTGGTGATTATAGCACCAGCAC
AAGTATCAGTGAGGACAGTTATGGCAGCTTCAGTGCTAATGGAAGCAGGCCTCACATGTCAAAG
GATGTGAGATGGGGAGCCATTAGGCGCATGGCTATCCAACAAGGGAGCTTGGGATTGAAGAACT
TCAAGCTTATCAAACAACTTGGTTGTGGGGACATTGGCACTGTTTATTTGGCTGAGTTAGTGGGC
TCAGACTGCATGTTTGCATTGAAGGTTATGGACATTGAGTACCTCATAAGCAGAAAGAAAATGCT
CAGAGCACAAACTGAGAGGGAGATACTGCAAATGCTCGACCATCCATTCCTTCCAACTCTTTATT
CTCATTTCACGACGGACAACCTTTCTTGTCTGGTAATGGAGTTCTGCCCTGGTGGTGACCTACATG
TCCTTAGGCAGAAACAACCTACCAAAACCTTTTCGGAAGCAGCTGCAAGGTATGAACTTATAGT
CATGTTATCGATGTGCTATCATTATGCACTAATACACATGTATCTGTCATGATTGAGCTGCTTTACC
AAGAACTAAAAACGGTGACTATATAACCTCGCTGTGTTTGGTACCAGATAGATGTTTTTTGTCCTG
TGATAGTATATCCCGCTATTGTAATCCTCCTGTGATAGTATATCCCGCTATTGTAATCCAATATTTTA
CAATTTTTTTATATAGGTTGTATTTTGCTAGTGAGTTATTGTAAATTAAGAGAATTTACATTTTATTTA
TATGTTTCATTTTAGTACTGAGCTATTGTAACTCGAGAAATCTACATTTTGTAGGTTTTATGTCGCT
GAAGTCCTCTTAGCCTTGGAGTATCTCCATATGTTGGGGGTCATATACCGTGATCTGAAGCCAGA
GAACATACTTGTTCGTGAAGATGGGCACATCATGCTCTCAGATTTTGATCTGTCTCTGAGGTGCTC
AGTGAGCCCAATGCTTGTGAGAACATCATCAGTAGGCAGAGATGAGCCCAGTAGGCCTTCTGGT
CCCTGTGCACAAAGCTGCATTGATCCCCTGTGTATCCAGCCATCCTGGAGCAATTCGTCCTGCTT
CACGCCCCGTTTGGTTTCGTCTACACCATCAAGGACACGGAGGCCTAGAGCTGAGCCCCTGAAG
AAACCATCACTTCCACAGCTCGTTGTTGAGCCCACGGAAGCAAGATCAAATTCATTTGTTGGGA
CCCATGAATATCTTGCCCCAGAGATCATTCGAGGAGATGGCCATGGTAGCTCGGTGGATTGGTGG
ACTCTTGGAATCTTCCTTTACGAATTACTCTACGGTAAGACACCATTCAAGGGACCTGGCAATGA
GGAAACACTCTCAAATGTGATCTCACAGGGCTTGAAGTTCCCTGACAACCCAGCTGTAAGCTTC
CACGCACGAGATCTTATCAGAGGGCTGCTTGTGAAAGAGCCAGAGTGCCGGCTCGGCTCGTCGA
GAGGAGCCGCTGAGATTAAACGGCATCCCTTCTTCGAAGGCCTAAACTGGGCCTTGATCCGGTG
GACTGCGCCACCAGAGACCCCGAAGAACATTGATACTGCAGCAGCACTCGCGACACGCAAGAA
AAAGGAAGGCAAATGTCTGGAATTCCGGCTGAATGGCGACGACATCGAGTTCGAGCTCTTTTAG 。

Wild-type sequence of *Dw3*:
ATGCTCGTCGGCACGCTCGGCGCGCTCGTCCACGGCTGCTCGCTCCCCGTCTTCCTCCGCTTCTT
CGCCGACCTCGTCGACTCCTTCGGCTCCCACGCCAACGACCCGGACACCATGGTCCGCCTCGTC
GTCAAGTACGCCTTCTACTTCCTCGTCGTCGGAGCCGCAATCTGGGCGTCCTCATGGGCAGGTAA
CCAACGTTATTCCTCCTCCTCCTCCTCTTCCTCCTCCCGGCACTGCTGCTCGCGTCGCGAATTGTC
TGTCGATTTGGATTGGATGGCGAATCACATCAGTCGCTCAATCTTCATGGCCCATGGCTAGCAAT
GAGATCGACCTTCGAATCCCTCGCTTGCAGAGATCTCCTGCTGGATGTGGACCGGCGAGCGGCA
GTCGACGCGGATGCGGATCCGGTACCTGGACGCGGCGCTGCGGCAGGACGTGTCCTTCTTCGAC

ACCGACGTGCGCGCCTCGGACGTCATCTACGCCATCAACGCGGACGCCGTGGTGGTGCAGGAC
GCCATCAGCGAGAAGCTGGGCAACCTCATCCACTACATGGCCACCTTCGTGGCGGGCTTCGTCG
TGGGCTTCACCGCCGCCTGGCAGCTGGCGCTCGTCACGCTCGCCGTCGTGCCGCTCATCGCCGTC
ATCGGGGGGCTCAGCGCCGCCGCGCTCGCCAAGCTCTCCTCCAGGAGCCAGGACGCGCTGTCG
GGCGCCAGCGGCATCGCGGAGCAGGCGCTCGCGCAGATACGGATCGTGCAGGCCTTCGTCGGC
GAGGAGCGCGAAATGCGGGCGTACTCGGCGGCGCTGGCCGTCGCGCAGAAGATCGGCTACCGC
AGCGGCTTCGCCAAGGGGCTCGGCCTCGGCGGCACCTACTTCACCGTCTTCTGCTGCTACGGCCT
CCTGCTCTGGTACGGCGGACACCTCGTCCGCGGCAACCACACCAACGGAGGGCTCGCCATCGC
CACCATGTTCTCCGTCATGATCGGCGGGCTGTAAGATGATCAGTTTCTCCCGGGCTCTCCTGTTCT
TCCGTCATGACACAGCATGTACTACGTACGCTTACTGGTCTGTGTCTGTGTGTGTGTGGATCGCGT
GCGTCCAGGGCCCTCGGGCAGTCGGCGCCGAGCATGGCCGCGTTCGCCAAGGCGCGCGTGGCG
GCCGCCAAGATCTTCCGCATCATCGACCACAGGCCGGGCATCTCCTCGCGGGACGGCGAGGAC
GGCGGCGGCGTGGAGCTGGAGTCGGTGACGGGGCGGGTGGAGATGAGGGGCGTGGACTTCGC
GTACCCGTCGCGGCCGGACGTCCCCATCCTGCGCGGCTTCTCGCTCAGCGTGCCCGCCGGCAAG
ACCATCGCGCTGGTGGGCAGCTCCGGCTCCGGGAAGAGCACGGTGGTGTCGCTCCTCGAGAGG
TTCTACGACCCCAGCGCAGGTATACATAGTACGCTACCAATTCTAGCTTTAGCGCATTGATTAATT
AGTGTTGGAGTTCACTTGCTTGCCAATTGCCATTGCCATCACACATCAGCAGCTACCATACATTGC
CAACTGCCATTGCTGCTGCCTTGCTGGGTGGTTAGTAGGGGAAGAAGCTTCCACTGTAGCAGGA
GTACATTGCAAACAGGAAGTGAATTTTGCAGTGGGAAATGAAGAAGTGAATGCTTGGAGCAGA
GCTGGCCGGCCTCATGGGCTGCTTACCTACTAGCTAGTCAACCAAGCATCCTGTTTCTTCCTTGTT
TATGGTCATGCATTCACACAGCTAGAACTAGAAGAGCTAGCCTTGTTTAGTTTCTAGAAAATTTTG
TAAAATTTTTAAGTTCCCTGTCACATCGAATCTTTAGACGCATGCATGGAGTATTAAATATAGACG
AAAATAAAAACTAATTGCACAGTTTGGTCGAAATTGACGAAACGAATCTTTTGAGCCTAATTAGT
CCATGATTGGACAATATTTGTCAAATACAAACGAAAGTGCTGCCGTATCGATTTTGCAAAGTTTTT
CGGAACTAAACAAAGCCTGGTGCCTGCAACGCGAGACAAAGAAAACTATTTGCCTGGCAAGAT
GCCACTATTGCACATGCATGCCACTCTTTGAGCCTTGACCGACTGACTGACTACTCAGAGTAGGA
GTGGTTCAATTGTATTGACATGTAGTAGGAGTACTCGTATGCTATAGTAGTCCTGTAGTTTTTTCAA
ACAAAAAAAAAAGAGAAAGAAAGAAATGAAGTCTGAAATTTGTTGGTTTTGGCAGGGCAAATC
TTGCTGGACGGGCATGATCTCAAGTCGCTGAAGCTCCGGTGGCTCCGGCAGCAGATTGGTCTGG
TGAGCCAGGAGCCGACGCTGTTCGCGACGAGCATCAAGGAGAACCTGCTGCTGGGGCGGGACA
GTCAGAGTGCGACGCAGGCCGAGATGGAGGAGGCCGCCAGGGTGGCCAACGCGCACTCCTTCA
TCGTCAAGCTCCCCGACGGCTACGACACGCAGGTCCGTATCGTATAGCTAGCTCCTAGCTGCACT
GTGCTGCCCTTTTCTTGCTCGCCACCGTTGCTGCCTGACATGTTTGTGCCTGTTGTTCTCCAACCC
ATTTGTCAGTGTCTAGACCACCATGCCTGCCTGCCTAGCTACTCGCTGCTCCCCGTCTCCTTCCTT
TCGCTACCCTGCCTTTTTTCTCATCTGCATGCAGCCTTTTTCTTTTCTCCATGTCATGAGAGGATGC
AGCATGTTGCATCATGTCATCCATCTCCATTCATGCGTCGCGCTCGTGCCTGCCCGGGGTAGCCTC
AGATCAGATACTGTTTGTGTTTGTTAGGCCGCGGCAGCGGCACATGAGCAGCTCATGACAGGCA
GCACCACCAACGCCATGTCCGCCATGGAGGTGTCGGGTCCATGTCCATTCCATCACAGCACGGC
ACTACTCATCCATCACATAGATACCAGTAGACAGACACTAGTACTAGTACTAGAACTAGGGATGG
GAGAGCAATTGCTTGTTAGGAGCCTGTTGCTATCACGATTGCTGTTGTGGGTTACCAACAAGTAA
CATGCCAGGGATGCTTTGCTATCACACACAGGACAGGAGAGGTCCCTTTCTCAACACGAAACTC
TACTAGCAGTTTAGCACTTGCTGCTGAGTGCTGAGAGCAGAGGGCAGAGGGAGATGAATGGATG
GTGAGCAGCTAGAGGAAGAGCCAGAAAAGGTTAATAACAATAGTAAAAAGATTAAGAATCAAC
CTGGGGTACGTAGAAAGAGGTAGAATTCCTAAGAATAATATAGGAGTGGGAGTGGAACAGAAC

AAATTCCAAGCTGGTATTTTTGTCAGGAATGTCAAGTTGATTTGATCCCAGTGCAAGCAAGAATT
ATCAATCACCCATCCTGTCTGTACAATCCAGCTCTTGCTACTCTACTTAGCACTACTGTGCTACTA
GTGGTAGGTATCTTCCACTTCTCTTAATATAATCTGTCATGAGAGAAAGAGAGTCAGACAAGCCC
ATGCTGCTGCTTATTTTAATCACTGTCAGTGGCAGGCAGTCTGGTTTGTTAATAACATCTGGGAAG
GGTTTAATCAAACCAGATCAAATCTAATGAAATCTAAGAGGTCACATGGGATATGGGCCACATAG
AGCAGGGCCTTGTTTAGTTCCCAAAAAATTTTACAAAATTTTTTAGATTCTCCATCACATCGAATC
TTTAGACACATGCATGAAGTATTAAATATAGATAAAAATAAAAACTAATTGCACAGTTTGGTCGA
AATTGACGAGACGAATCTTTTGAACCTAGTTAGTCTTTGATTGGATAATATTTGTCAAATACAAAC
GAAAAAGCTACAGTGTCGATTTTGCAAAATATTTTGGAACTAAACAAGGCCCAGCTCGCCTTCC
CACACTCCCATGACTGATTCCTTGCTAAGCAATGCTCGCATGCCCATGCATCATCCCTGGTCAAA
CACTCTGCACCATCATAAGACTAGTATTAACAAATGATTTCATTTTTGTTGTTATTAATTATGCTGG
AGGAGTGGTACTATTTTATTATATTTGATGAAAAACTTGGCAGTCAAAGTCAACCCGTTTGTTTGA
CACTGCGTGCATGGCCGGTGCAGGTTGGGGAGCGCGGCCTGCAGCTCTCCGGCGGGCAGAAGC
AGCGCATCGCCATCGCCCGCGCCATGCTCAAGAACCCTGCCATCCTGCTGCTGGACGAGGCTAC
CAGCGCGCTCGACTCCGAGTCGGAGAAGCTCGTGCAGGAGGCGCTGGACCGCTTCATGATCGG
GCGCACCACCCTGGTGATCGCGCACAGGCTGTCCACCATCCGCAAGGCCGACGTCGTGGCCGTG
CTGCAGGGCGGCGCCGTCTCCGAGATGGGCACCCACGACGAGCTCATGGCCAAGGGCGAGAAC
GGCACCTACGCCAAGCTGATCCGCATGCAGGAGCAGGCGCACGAGGCGGCGCTCGTCAACGCC
CGCCGCAGCAGCGCCAGGCCCTCCAGCGCCCGCAACTCCGTCAGCTCGCCCATCATGACGCGC
AACTCCTCCTACGGCCGCTCCCCCTACTCCCGCCGCCTCTCCGACTTCTCCACCTCCGACTTCAC
CCTCTCCATCCACGACCCGCACCACCACCACCGGACGATGGCCGACAAGCAGCTCGCGTTCCG
CGCCGGCGCCAGCTCCTTCCTCCGCCTCGCCAGGATGAACTCGCCCGAGTGGGCCTACGCGCTC
GTCGGCTCCCTGGGCTCCATGGTCTGCGGCTCCTTCAGCGCCATCTTCGCCTACATCCTCAGCGC
CGTGCTCAGCGTCTACTACGCGCCGGACCCTCGCTACATGAAGCGCGAGATCGCCAAGTACTGC
TACCTGCTCATCGGCATGTCCTCCGCGGCGCTGCTGTTCAACACGGTGCAGCACGTGTTCTGGGA
CACGGTCGGCGAGAACCTCACGAAGCGTGTGCGCGAGAAGATGTTCGCCGCCGTGCTCCGCAA
CGAGATCGCCTGGTTCGACGCCGACGAGAACGCCAGCGCGCGCGTCGCCGCCAGGCTCGCGCT
CGACGCCCAGAACGTGCGCTCCGCCATCGGGGACCGTATCTCCGTCATCGTCCAGAACTCGGCG
CTCATGCTCGTCGCCTGCACCGCGGGCTTCGTCCTCCAGTGGCGCCTCGCGCTCGTGCTCCTCGC
CGTCTTCCCGCTCGTCGTCGGCGCCACCGTCCTGCAGAAGATGTTCATGAAGGGCTTCTCGGGG
GACCTGGAGGCCGCGCACGCCAGGGCCACGCAGATCGCGGGCGAGGCCGTCGCCAACCTGCG
CACCGTGGCGGCGTTCAACGCGGAGCGCAAGATCACGGGGCTCTTCGAGGCCAACCTTCGCGG
CCCGCTCCGGCGCTGCTTCTGGAAGGGGCAGATCGCCGGGAGCGGCTACGGCGTGGCGCAGTT
CCTGCTGTACGCGTCCTACGCGCTGGGGCTCTGGTACGCCGCGTGGCTAGTGAAGCACGGCGTC
TCCGACTTCTCGCGCACCATCCGCGTGTTCATGGTGCTCATGGTGTCCGCCAACGGCGCCGCCGA
GACGCTGACGCTGGCGCCGGACTTTGTCAAGGGCGGGCGCGCGATGCGGTCCGTGTTCGAGAC
CATCGACCGGAAAACGGAGGTGGAGCCCGACGACGTGGACGCGGCGCCGGTGCCGGAGCGGC
CCAAGGGCGAGGTGGAGCTGAAGCACGTGGACTTCTCGTACCCGTCGCGGCCGGACATCCAGG
TGTTCCGCGACCTGAGCCTCCGGGCGCGCGCCGGGAAGACGCTGGCGCTGGTGGGTCCGAGCG
GGTGCGGCAAGAGCTCGGTGCTGGCGCTGGTGCAGCGGTTCTACGAGCCCACGTCCGGGCGCG
TGCTCCTGGACGGCAAGGACGTGCGCAAGTACAACCTGCGGGCGCTGCGGCGCGTGGTGGCGG
TGGTGCCGCAGGAGCCGTTCCTGTTCGCGGCGAGCATCCACGACAACATCGCGTACGGGCGCG
AGGGCGCGACGGAGGCGGAGGTGGTGGAGGCGGCGACGCAGGCGAACGCGCACCGGTTCATC
TCGGCGCTGCCGGAGGGCTACGGGACGCAGGTGGGCGAGCGCGGGGTGCAGCTGTCGGGCGG

GCAGCGGCAGCGGATCGCGATCGCGCGCGCTGGTGAAGCAGGCGGCCATCATGCTGCTGGA
CGAGGCGACCAGCGCGCTGGACGCCGAGTCGGAGCGGTGGCTCTTCGAGGCCAACCTTCGCGG
CCCGCTCCGGCGCTGCTTCTGGAAGGGGCAGATCGCCGGGAGCGGCTACGGCGTGGCGCAGTT
CCTGCTGTACGCGTCCTACGCGCTGGGGCTCTGGTACGCCGCGTGGCTAGTGAAGCACGGCGTC
TCCGACTTCTCGCGCACCATCCGCGTGTTCATGGTGCTCATGGTGTCCGCCAACGGCGCCGCCGA
GACGCTGACGCTGGCGCCGGACTTTGTCAAGGGCGGGCGCGCGATGCGGTCCGTGTTCGAGAC
CATCGACCGGAAAACGGAGGTGGAGCCCGACGACGTGGACGCGGCGCCGGTGCCGGAGCGGC
CCAAGGGCGAGGTGGAGCTGAAGCACGTGGACTTCTCGTACCCGTCGCGGCCGGACATCCAGG
TGTTCCGCGACCTGAGCCTCCGGGCGCGCGCCGGGAAGACGCTGGCGCTGGTGGGTCCGAGCG
GGTGCGGCAAGAGCTCGGTGCTGGCGCTGGTGCAGCGGTTCTACGAGCCCACGTCCGGGCGCG
TGCTCCTGGACGGCAAGGACGTGCGCAAGTACAACCTGCGGGCGCTGCGGCGCGTGGTGGCGG
TGGTGCCGCAGGAGCCGTTCCTGTTCGCGGCGAGCATCCACGACAACATCGCGTACGGGCGCG
AGGGCGCGACGGAGGCGGAGGTGGTGGAGGCGGCGACGCAGGCGAACGCGCACCGGTTCATC
TCGGCGCTGCCGGAGGGCTACGGGACGCAGGTGGGCGAGCGCGGGGTGCAGCTGTCGGGCGG
GCAGCGGCAGCGGATCGCGATCGCGCGCGCTGGTGAAGCAGGCGGCCATCATGCTGCTGGA
CGAGGCGACCAGCGCGCTGGACGCCGAGTCGGAGCGGTGCGTGCAGGAGGCGCTGGAGCGCG
CCGGGAACGGCCGCACCACCATCGTGGTGGCGCACCGGCTGGCCACGGTGCGGAACGCGCACA
CCATCGCCGTGATCGACGACGGCAAGGTGGTGGAGCAAGGGTCGCACTCGCACCTGCTCAAGC
ACCATCCCGACGGGTGCTACGCGCGGATGCTGCAGCTGCAGCGGCTGACAGGCGGTGCCGCGC
CCGGGCCGCCGCCGTCGTCGTCCAACGGGGCCGCCGCGTAGGATGGATGGATGGATCATGGATG
AGTTTGGTTCCTTGAGAGATTGATGGATGAGGAAGCTGAAGCTCCGGAGGGAATGATGGTACTC
CATGATCGCAACAAGGGGAAAAGAAAAAAAGAAGCAGAAAACACGGTGGTTCATATGATTGTA
CAATTTGATGATGATCTCTTTGAGTTGAGGTTTTAGGATGATGTAAACTTTCACATCTTTTTTTTTTT
GACTCTTTTTGCTTCTCATTCTTCTTGTTTCTCATCTGCTAGCTGGGACTGAGATGTGGAACAGATA
GAAGCAACAGCATCTATCTAAATACAGTGCCAGAGATGGGAATTGATCCTTCCTTGTAAGTAGAC
GATGGCTAGCTCCCAACTTCCCAAGGAAAAGGAACCCAGATCATATGTACTACTGTACTTATACT
GTATTAGTTAGCCTCTTATATACACCATATATAGCAGATTTACTGCTTATTTGCAATAGCAGCAACA
GATAGGGACCAGAAAAATGAAGGTAGTGCATGAATGATTGCTACTATCGGTGAGTACTCCCTCC
GTATCAAATTACAAGTCATTTTAAGAATCTTGGAGAGTCAAAGATTTTTAAGTTTGACCAAATTTA
TATAGTAAAACAATAATATTTTTGGTATCAACTAAG
